# Cross-Cultural Translation and Adaptation of the Consumer Ear Disease Risk Assessment (CEDRA) Questionnaire in Danish

**DOI:** 10.3390/audiolres13060075

**Published:** 2023-11-02

**Authors:** Lene Dahl Siggaard, Henrik Jacobsen, Dan Dupont Hougaard, Mina Shereen Khaled, Morten Høgsbro

**Affiliations:** 1 Department of Otolaryngology, Head and Neck Surgery, and Audiology, Aalborg University Hospital, 9000 Aalborg, Denmark; heja@rn.dk (H.J.); shereenkma@gmail.com (M.S.K.);; 2 Department of Clinical Medicine, Aalborg University, 9000 Aalborg, Denmark

**Keywords:** hearing loss (MeSH term), ear disease, questionnaire translation, risk assessment, screening tool

## Abstract

This study aimed to cross-culturally translate and adapt the Consumer Ear Disease Risk Assessment (CEDRA) questionnaire into Danish for remote ear, nose, and, throat assessments in adult, first-time hearing aid users when used in conjunction with audiometric measures and visual images of the tympanic membrane. Employing field-specific guidelines, the tool underwent a rigorous translation process. This was succeeded by field testing via cognitive debriefing with 30 intendent respondents and a pilot test involving 600 adult, potential first-time hearing aid users from 2020–2022. Test–retest reliability analysis in 113 respondents revealed high consistency and reproducibility, with most items showing Spearman’s correlation coefficients of 0.82 or higher and a Pearson’s correlation of 0.92 for the total score. The tool demonstrated moderate discriminative ability in identifying individuals at high and low risk of complicated hearing loss and targeted ear diseases, supported by an area under the curve of 0.82 on the receiver operating characteristics curve. Our findings suggest that the Danish-translated version of CEDRA is a reliable and effective screening instrument when used with audiometry and tympanometry, warranting further validation in a larger population.

## 1. Introduction

The Danish hearing healthcare system has faced political criticism for its structural inefficiency and delayed initiation of proper hearing rehabilitation for individuals with hearing loss (HL) [1]. Issues include prolonged diagnosis, long wait times for treatment in public audiological clinics, and the repetition of audiometric tests before treatment is initiated [1]. Delayed or inadequate hearing rehabilitation for individuals with age-related HL can increase the risk of dementia, anxiety, and social distancing and negatively impact their quality of life [2,3]. It is estimated that between 500,000 and 800,000 Danish-speaking individuals experience varying degrees of HL, with approximately 300,000 of them having been prescribed a hearing aid (HA). Additionally, there are an estimated 46,000 new cases a year [1]. Given the projected increase in the population of individuals of 60 years and over [4], the prevalence of age-related HL is expected to rise, thereby elevating the demand for hearing rehabilitation services. However, current organizational challenges within the Danish hearing rehabilitation system may impede its capacity to meet this escalating demand, potentially exacerbating the detrimental consequences of delayed treatment in the future.

Currently, adult Danish-speaking individuals who are potential first-time users of HAs require a physical ear, nose, and throat (ENT) specialist assessment (PESA) screening conducted in-person by private ENT specialists before initiating treatment. Patients with mild or moderate HL have the option to acquire HAs through either private or public audiological clinics. In contrast, those with severe or severely asymmetrical HL, or serious ear disorders, require specialized assessment and treatment at a public audiology or otology hospital department [5]. In an effort to minimize diagnostic and treatment delays, a digital and remote ENT specialist assessment (RESA) screening protocol was formulated and evaluated in a randomized clinical trial between 2021 and 2022 in the north Denmark region. The trial involved 751 adult, potential first-time HA users [6]. The primary objective of the RESA screening protocol was to efficiently identify patients with complicated HL or serious ear disorders who require care. Concurrently, the RESA screening method sought to obviate the need for individuals with mild to moderate HL to visit private ENT specialist clinics before acquiring HAs. This approach aimed to avoid redundant audiometric tests while preserving a high level of ENT specialist screening accuracy, as well as maintaining patient-reported treatment benefits and satisfaction. RESA screening was executed in two phases: initially, a standardized examination was conducted by certified technical audiologists in either private or public audiological clinics. This examination encompassed a patient-reported medical history focused on ears and hearing, audiometric testing including air and bone conduction thresholds, a speech discrimination test, acoustic reflex tests, a standard 226 Hz tympanometry, and the capture of still images of the tympanic membranes via video otoscopy. Subsequently, ENT specialists reviewed these results digitally, remotely, and asynchronously, without direct physical or telemedical interaction with the participants. Based on the severity of the HL, the specialists then referred the participants to the appropriate treatment regime. The results from the trial indicated that RESA screening demonstrated markedly superior accuracy in diagnosing complicated HL and serious ear disorders compared to the traditional PESA screening. Specifically, RESA screening exhibited a sensitivity of 85% and a specificity of 97%, in contrast to the PESA method, which showed a sensitivity of 20% and a specificity of 100% [6]. The notably lower sensitivity observed in PESA screening could be attributed to factors such as abbreviated consultation durations and the suboptimal quality of audiometric examinations, which may have been performed by clinical staff rather than certified technical audiologists in private ENT specialist clinics.

Given the absence of direct patient–physician interaction in the RESA screening process, there was a need for an assessment tool capable of screening for a range of targeted ear diseases (TEDs) associated with HL. These included diseases related to the external auditory canal such as exostosis, otitis externa, and cholesteatoma of the external auditory canal; conditions of the middle ear like cholesteatoma, otosclerosis, and perforation, or retraction of the tympanic membrane, infection, and otitis media; abnormalities in retro-cochlear structures such as vestibular schwannoma, tinnitus, and otogenic vertigo; and cerebral issues such as infection, tumors, head trauma, and vascular disorders or neurological disorders. The multi-complaint, 15-item Consumer Ear Disease Risk Assessment (CEDRA) questionnaire was identified as a suitable tool for this purpose, as it screens for 104 TEDs related to HL among potential first-time HA users and aligns well with the construct of interest. The types of TEDs identified in CEDRA encompass conductive/middle ear or mastoid diseases, sensorineural or labyrinthine conditions, those related to the eighth nerve, or those situated in the posterior fossa [7]. Since a Danish version of the CEDRA did not exist, and its utility had not been previously studied in Denmark, a cross-cultural translation and adaptation process was initiated. The objective of this study was to document the process of translating and culturally adapting the Danish version of the CEDRA. The intent is for this version to be employed by Danish-speaking ENT specialists within a digital RESA framework, complemented by additional audiometric evaluations, such as audiometry and tympanometry, as well as visual images of the tympanic membranes. Taken together, these various data components will function as a comprehensive screening tool for identifying complicated HL and TEDs in adult, potential first-time HA users who require specialized assessment and treatment at public audiology or otology hospital departments.

## 2. Materials and Methods

To facilitate remote screenings by ENT specialists for potential first-time HA users without direct consultations, the RESA screening package needed to incorporate a risk assessment tool. This tool would screen for various historical or current TEDs associated with HL, alongside audiometric and tympanometric measures, as well as visual images of the tympanic membranes. To this end, an expert committee and translational team was formed, consisting of professional translators, linguistic experts, healthcare practitioners specializing in audiology and otology, as well as members of the target population. This committee aimed to ensure a robust cross-cultural translation and adaptation of the questionnaire into the target language. The committee identified the questionnaire’s intended purpose, the relevant hearing-associated construct, its psychometric properties, and its feasibility, all in line with established guidelines for good practice in translating and adapting hearing-related questionnaires across languages and cultures [8]. A literature review identified 155 unique questionnaires in the English-language literature related to audiology and neurotology for adult populations [9]. Among these, 15 were multiple complaint questionnaires assessing symptoms and quality of life concerning various ear disorders. CEDRA emerged as the sole tool that closely aligned with the construct of interest and was conceptually relevant across both the source and target countries where the questionnaire would be deployed. Furthermore, the committee ascertained that the questionnaire in its source language exhibited satisfactory psychometric properties and was feasible in terms of completion time, cost, and comprehensibility [10].

### 2.1. The Consumer Ear Disease Risk Assessment (CEDRA) Questionnaire

CEDRA was developed in response to two significant regulatory shifts in the United States (U.S.): the discontinuation of the medical waiver enforcement option for providing HAs by the U.S. Food and Drug Administration in 2016 [11] and the enactment of the Over-The-Counter Hearing Aid Act of 2017 [12]. The primary objectives of CEDRA were twofold: to offer adult first-time HA users a self-screening tool for identifying 104 different TEDs and to assist clinicians in providing tailored hearing rehabilitation advice [7,10]. The instrument comprises 15 items encompassing a range of conditions related to ear health, hearing, balance, and tinnitus, as well as co-occurring symptoms and general health indicators associated with HL. CEDRA scores range from 0 to 28 and are calculated based on the respondents’ answers. Higher scores signify an elevated risk of one or more among 104 TEDs that would necessitate medical evaluation prior to HA acquisition. It is recommended that individuals with CEDRA scores of four or above seek medical consultation. In its original format, the tool was found to be readily comprehensible to its target audience and could be completed in approximately 10 min [10]. Prior to undertaking the cross-cultural translation, permission for both usage and translation of the questionnaire was sought and obtained from the developers of the source-language instrument.

### 2.2. Forward and Backward Translation

The translation process strictly adhered to the good practice guidelines for translating and adapting hearing-related questionnaires [8]. To this end, two independent translators—both native speakers of Danish and bilingual—were recruited: one with a medical background and one with linguistic expertise. Both translators were provided with standardized instructions, formulated by the expert committee, aimed at ensuring conceptual, item, and semantic equivalence. They were also guided to use non-technical language to enhance the tool’s comprehensibility for its intended audience. Each translator then independently undertook the task of forward-translating CEDRA into Danish. Upon completion of the forward translations, the expert committee reviewed them to identify and reconcile any discrepancies, thereby producing a unified consensus version suitable for backward translation. This consensus version was then independently back-translated into the source language by two professional bilingual speakers residing in Denmark and familiar with Danish culture. By comparing these backward translations with the original source-language questionnaire, and also against the forward translations, the committee was able to resolve any remaining inconsistencies. The end result was a consensus version of the translated questionnaire that maintained conceptual, semantic, and operational equivalence with the original CEDRA instrument. The Danish version was dubbed ‘RiHab’, which is the Danish equivalent of ‘CEDRA’ (see the Supplementary Material).

### 2.3. Field Testing

#### 2.3.1. Cognitive Debriefing

The methodology of cognitive debriefing was employed to evaluate the feasibility of the consensus version. This was tested on 30 respondents, comprising 55% males, with an average age of 64 years of whom 55% were first-time HA users. These respondents were representative of the target demographic of adults reporting subjective HL. Each respondent participated in a comprehensive, semi-structured, individual cognitive interview conducted by an interviewer with specialized medical knowledge in otorhinolaryngology. These interviews were recorded and subsequently transcribed verbatim by a medical secretary. The interviewer compiled two summary reports that highlighted recurring issues and key insights mentioned by the respondents during their interviews. The first report synthesized the findings from the initial 12 interviews, and after appropriate adjustments were made to the questionnaire by the expert committee, a second report was generated based on an additional 18 interviews to identify any lingering misunderstandings.

Following a rigorous review of the first summary report, minor yet essential modifications were integrated into the consensus version of the questionnaire before subjecting it to further testing with the remaining 18 respondents. Subsequent revisions, guided by insights from the second summary report, were incorporated to ensure the semantic equivalence, accessibility, and appropriateness of language in the new version. The final steps included formatting and proofreading, culminating in the release of the finalized version. The objective of the present study was to offer the questionnaire in either a paper-based or digital format, or both, depending on the participants’ preference, and to streamline data collection. Both formats were considered beneficial for future use in comparable settings. Comprehensive documentation of the entire translation and cross-cultural adaptation process was completed and archived.

#### 2.3.2. Pilot Testing

To assess the efficacy of RiHab in the identified patients with TEDs associated with HL among prospective first-time HA users, a screening accuracy analysis was conducted on 600 intended participants between 2020–2022. The median age of the respondents was 64 years, with an interquartile range of 54 to 71 years, and 52% were female. This evaluation involved juxtaposing the participants’ RiHab scores against a ‘gold standard’ assessment administered by ENT specialists within the realms of medical audiology and otology. All participants provided written informed consent prior to engagement in the study. The participants completed the RiHab questionnaire before undergoing audiometric and tympanometric examinations conducted by certified, experienced technical audiologists. This was followed by a 30-min consultation and objective examination, including oto-microscopy, performed by an ENT specialist. Unaware of the RiHab scores, the ENT specialists classified the participants into one of two diagnostic categories based on the presence or absence of TEDs.

The first diagnostic group comprised participants exhibiting either objectively normal hearing, characterized by an air conduction (AC) pure-tone average (AC-PTA) hearing level of 20 dB or better, or those with HL of varying magnitudes attributable to age or noise exposure. These ranged from mild HL (AC thresholds of 21–40 dB) and moderate HL (AC thresholds of 41–60 dB) to severe or complicated HL exceeding AC thresholds of 61 dB and/or showcasing asymmetry in the average AC thresholds at frequencies of 500, 1000, 2000, and 4000 Hz (AC-PTA-4) greater than 30 dB between ears and/or a discrimination score (DS) of 20% or more between the two ears [5]. Importantly, this first group did not include participants suspected of having or diagnosed with any TEDs, regardless of the presence or severity of HL.

The second diagnostic group included participants identified with one or more TEDs, irrespective of the presence or severity of HL. Elevated RiHab scores were anticipated in this group.

The participants were recruited either during their in-person visits to the Department of Otolaryngology, Head and Neck Surgery, and Audiology at Aalborg University Hospital or via the north Denmark region’s Facebook page [6]. The inclusion criteria stipulated that the participants had to be 18 years of age or older, report subjective HL, and have no prior experience with HAs. The exclusion criteria encompassed a lack of proficiency in the Danish language, the presence of severe dementia, or other cognitive impairments that could hinder informed consent and successful completion of the RiHab questionnaire.

### 2.4. Psychometric Evaluation

Although not explicitly stipulated in the minimum good practice guidelines for the translation and adaptation of hearing-related questionnaires, psychometric evaluation of the target questionnaire is generally recommended [8]. In the case of a multidimensional questionnaire like CEDRA, item response theory (IRT) analysis can provide a robust approach to its translational and cross-cultural adaptation. IRT’s capability to detect differential item functioning ensures that items maintain consistent meanings across varied cultural or linguistic backgrounds, thereby identifying potential cultural biases. The calibration properties of IRT allow for a comparative analysis of item behaviors across different cultures, shedding light on possible translation or interpretation discrepancies. This rigorous methodology aids in enhancing questionnaires by selecting items that are both culturally relevant and informative. Additionally, IRT enables the harmonization of various questionnaire versions or languages, guaranteeing that scores are comparable [13]. Nevertheless, such an IRT analysis fell outside the purview of the present study.

#### Reliability

To assess the stability and consistency of the test scores over time, a reliability analysis was performed, which included both a test–retest and an estimation of the standard error of measurement (SEM). According to the existing literature, a sample size of 117 respondents is considered sufficient for a robust test–retest reliability analysis [14]. To account for a potential 20% drop-out rate, a total of 145 respondents were recruited. These respondents were patients at the Department of Audiology at Aalborg University Hospital in Aalborg, Denmark, and were enlisted during their on-site visits to the facility. In total, 113 test–retest questionnaires were completed and incorporated into the analysis. After providing written informed consent, the respondents received an email containing a link to the questionnaire. A second link to a follow-up questionnaire was sent three days after completion of the initial questionnaire. Data, including responses and RiHab scores, were securely stored in the REDCap^®^ system [15,16]. Questionnaires that were incomplete or were completed within less than three days or more than 14 days after receiving the initial link were excluded from the analysis.

### 2.5. Statistical Analysis

All statistical tests were executed using R statistical software, version 4.1.2 [17]. To assess the temporal reliability of RiHab, the Spearman’s correlation coefficient for each individual item in the test–retest evaluation was calculated. For the overall RiHab scores obtained in the test–retest, Pearson’s correlation coefficient was utilized, and the intraclass agreement correlation coefficient (ICC) was computed using a one-way random-effects model. The standard error of measurement (SEM) was ascertained based on the aggregate RiHab scores derived from the test-retest evaluation, utilizing the ‘SimplyAgree’ R package, version 0.1.2 [18].

For the evaluation of RiHab’s screening efficacy in a preliminary testing environment, a receiver operation characteristic (ROC) curve was generated. This curve incorporated two key metrics of diagnostic accuracy: sensitivity and specificity. Sensitivity denoted the proportion of respondents accurately classified as having one or more TEDs, while specificity indicated the proportion of respondents accurately classified as not having any TEDs. For the purpose of this analysis, all individual TEDs were consolidated into a single dichotomous variable, representing either the absence or presence of any TED. All derived metrics were reported with 95% confidence intervals (CIs) and a *p*-value of less than 0.05 was deemed statistically significant.

## 3. Results

### 3.1. Field Testing

#### 3.1.1. Cognitive Debriefing

A report on the first 12 respondents’ interviews indicated that RiHab was generally clear and comprehensible. Nevertheless, a few misunderstandings and misinterpretations of certain verbs and nouns used in the questionnaire were observed. For instance, the term “pus” in item: *“Have you ever noticed pus, blood or other active fluid discharge from your ear?”* was misconstrued as earwax by five respondents. As a result, a different word that more accurately represented the meaning of “pus” was adopted. Additionally, the term “Ménière’s disease” in item nine led to confusion with nine respondents unsure of their response as they were unfamiliar with the condition. To address this, the question was modified to *“Have you ever been told by a physician that you have Ménière’s disease? If you have never heard of Ménière’s disease, circle ‘No’”*. Another challenge arose with item 13b, where none of the 12 respondents could differentiate between *“pressure in the ear”*, *“fulness in the ear”*, and *“plugged feeling in the ear”* all linked to potential cooccurring tinnitus symptoms. Consequently, each term was supplemented with a clarifying description. Lastly, the self-scoring instructions on page three of the original CEDRA tool were found to be confusing by five respondents, leading to miscalculations of their RiHab scores. Hence, the expert committee undertook thorough revision and modification of the self-scoring instructions to enhance readability and comprehension. The modifications made to the scoring instructions were chiefly linguistic, aiming to provide clearer guidance for respondents when calculating their scores. In practice, this involved employing more comprehensive sentences and varied terminology to improve understanding.

Another 18 respondents completed the revised version, followed by individual interviews. The expert committee continued revising the questionnaire until no further changes were necessary or until the proposed modifications reverted to previous versions. A final report concluded that the changes had significantly reduced the previously reported misunderstandings. However, nine out of the 18 respondents still miscalculated their RiHab score. Furthermore, one patient found it inconsistent and confusing that responses to some items required circling, while others needed to be checked. To circumvent these potential issues and to respond to the respondents’ needs, a digital version was employed for further analysis where answers were consistently checked and the RiHab score was automatically calculated based on the given responses, requiring no further action from the respondent.

#### 3.1.2. Pilot Testing

**Figure 1:**
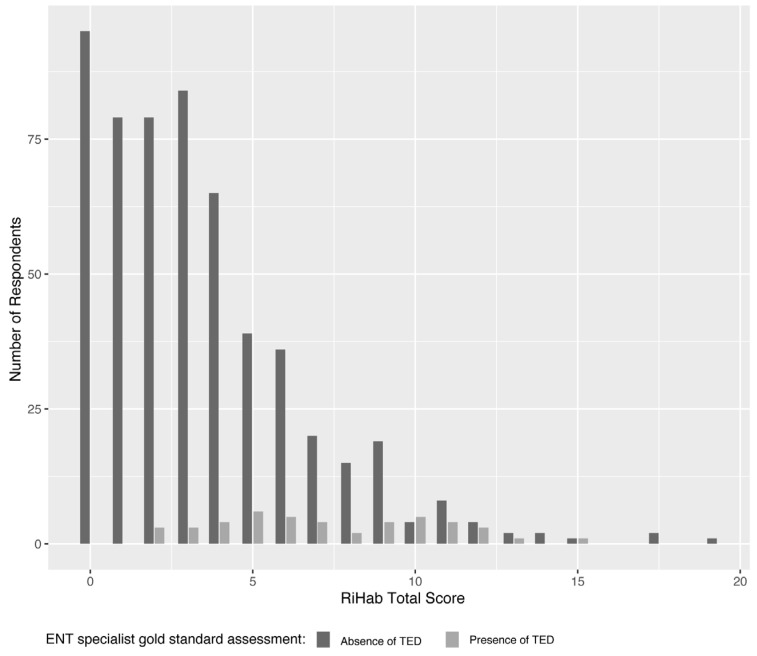
Distribution of RiHab scores among the 600 respondents stratified into two diagnostic groups: (1) respondents with the presence of TEDs (n = 45), and (2) respondents with the absence of TEDs regardless of HL severity (n = 555). Abbreviations: ENT: ear, nose, and throat; TED: targeted ear disease.

The screening efficacy of RiHab as a risk assessment tool for TEDs was evaluated in a cohort of 600 adults who reported subjective HL and were potential first-time users of HAs. ENT specialists, who were blinded to the RiHab scores, categorized the respondents into two diagnostic groups based on the presence or absence of one or more TEDs associated with HL. Out of the total, 555 respondents (93%) exhibited either normal hearing (n = 277, 46%) or varying degrees of age- or noise-induced HL—mild, moderate, or severe (n = 278, 46%)—but had no identified TEDs. Conversely, the remaining 45 respondents (7%) were diagnosed with one or more TEDs, such as cholesteatoma of the middle ear, cholesteatoma of the external auditory canal, otosclerosis, tympanic membrane perforation, otitis media with effusion, or eustachian tube dysfunction. Figure 1 depicts the distribution of RiHab scores across these two diagnostic groups. In the subset of respondents with TEDs, the total RiHab scores ranged from 3–15.

**Figure 2:**
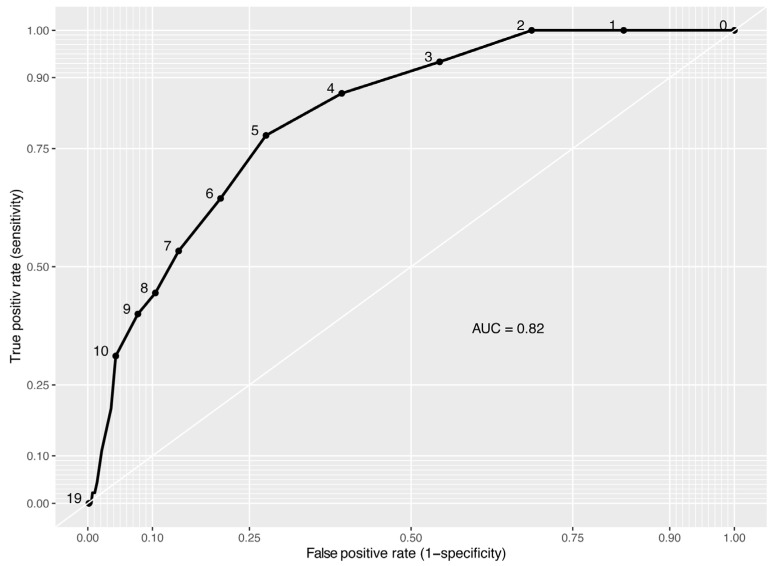
Receiver operating characteristic (ROC) curve for RiHab as a binary diagnostic indicator for targeted ear diseases. AUC indicates the area under the curve.

Figure 2 depicts the ROC curve for RiHab as a binary diagnostic indicator for the presence of one or more TEDs across all respondents. The area under the curve (AUC) serves as an index for evaluating the overall efficacy of RiHab as a binary classifier. AUC values range from 0.50 indicative of a performance no better than chance, to 1, representing perfect discriminatory ability. An AUC of 0.82 (95% CI: 0.76–0.87) was observed. The optimal score threshold for the tool is identified as the point on the curve situated closest to the top-left corner of the plot.

Table 1 provides a subset of data specifying sensitivity and specificity for different RiHab scores.

### 3.2. Reliability

Out of the 154 respondents initially recruited for the test–retest reliability analysis, 113 (73%) successfully completed the RiHab questionnaire twice within the prescribed timeframe of 3–14 days. The mean time to respond was 4.91 days (standard deviation (SD): 0.89), with a range from 4.0 to 7.4 days. The mean score for the initial RiHab test was 2.74, (SD: 2.65), spanning a range of zero to 12; for the retest, the mean score was 2.71, (SD: 2.78), with a range of zero to 14. Table 2 presents Spearman’s correlation coefficients for the individual item responses. The Pearson’s correlation coefficient for the aggregate RiHab scores in the test–retest analysis was 0.92 (95% CI: 0.85–0.93), *p* < 0.0001, indicating a high degree of correlation between the total scores in both tests. The agreement ICC was 0.90 (95% CI: 0.86–0.92) and the SEM was 0.90 (95% CI: 0.76–0.99) for the overall RiHAb scores in the test–retest.

**Figure 3:**
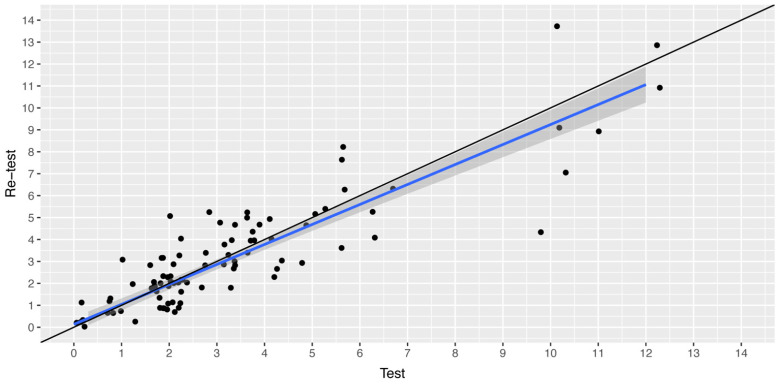
Scatter plot of the RiHab scores from the test–rest-analysis. The total RiHab scores are plotted along the *x*-axis against the retest total RiHab scores on the *y*-axis. The blue line is a linear regression and is in close approximation to the true diagonal line (black). The shades of gray indicate the 95% confidence interval for the regression line. The points have been jittered for visualization only.

Figure 3 illustrates a scatter plot of the RiHab scores from both the initial test and the retest.

## 4. Discussion

The aim of this manuscript was to conduct a cross-cultural translation and adaptation of CEDRA into Danish, in accordance with field-specific good practice guidelines [8]. A final digital version, equipped with an automatic scoring function and termed RiHab, was subsequently developed.

The majority of items displayed Spearman’s correlation coefficients equal to or greater than 0.70 in the test–retest analysis, indicating the consistent and reproducible performance of RiHab over time. Nonetheless, three items associated with changes in hearing and two items pertaining to ear fullness and blockage in relation to tinnitus exhibited coefficients of 0.5 or lower. This suggests that the responses to these items may be subject to temporal variability, possibly due to day-to-day fluctuations in the perception of HL and tinnitus symptoms. Despite these inconsistencies, the aggregate RiHab scores exhibited a high degree of correlation between the initial test and retest, as evidenced by a Pearson’s correlation coefficient of 0.92 and an agreement ICC of 0.90. Furthermore, the distribution of test–retest RiHab scores around a diagonal line on the scatterplot suggests strong overall consistency. Given the potential score range of 0–28, an SEM of 0.90 indicates a relatively low level of measurement error. This suggests that RiHab is generally a reliable instrument, although some minor remaining error persists. Consequently, a difference of one or two points in RiHab scores is likely indicative of a genuine variation in the risk of TEDs.

The screening efficacy of RiHab, as a risk assessment tool for TEDs, was assessed in a cohort of 600 adults with subjective HL who were potential first-time HA users. The participants were stratified into two diagnostic categories by ENT specialists based on the presence or absence of TEDs, independent of the severity of HL. The demographic characteristics of the study population, specifically in terms of age and gender, appeared to be reflective of the general population seeking HAs. The 7% prevalence of TEDs observed in our sample of 600 participants appears to be somewhat consistent with the 5% TEDs prevalence estimated for Danish-speaking adults with HL, as reported by the Danish National Board of Social Services in 2010. While this comparison is discussed in other sources, more recent national statistics are not available [6]. Remarkably, audiometric examinations revealed that 46% of respondents had objectively normal hearing, which suggests that subjective HL is prevalent but may not, in isolation, constitute a compelling indication for HA utilization. An AUC value of 0.82, as depicted in the ROC curve in Figure 2, denotes the relatively high discriminative capacity of the RiHab tool, although it is not perfect. This, however, underscores RiHab’s proficiency in effectively differentiating between the two distinct groups of adults with subjective HL—those with and without TEDs—regardless of their actual hearing capabilities or the severity of HL. Moreover, the sizeable sample of 600 participants furnished a robust statistical foundation for the RiHab screening accuracy analysis, which enhances the reliability of the AUC estimates and the generalizability of the findings.

One strength of the study lies in the systematic methodology employed, in line with established best practices for the cross-cultural translation and adaption of hearing-related questionnaires [8]. Also, the respondents completed the test–retest online, in their homes, thereby reducing the likelihood of physician or clinical staff influence. Although the study did not specifically evaluate the presence of assistance or susceptibility to acquiescence or social desirability biases, such risks are considered minimal due to the neutrality of the questions and the absence of extreme wording. While the three-day interval between the test and retest adheres to recommended guidelines [19], the potential for recollection bias should not be discounted.

The most favorable balance between RiHab’s sensitivity and specificity as gleaned from our data was 74% and 62%, respectively, and observed at a cut-off score of five. It should be noted, however, that the selection of an optimal cut-off score inherently involves a trade-off between the tool’s sensitivity and specificity. This balance may vary depending on the clinical or research objectives, as well as the associated costs of either delayed diagnoses—when the cut-off is set too high and patients with TEDs risk being overlooked—or unnecessary medical evaluations and hospital visits—when the cut-off is set too low, leading to an increase in false-positive diagnoses [10]. This is further corroborated by the distribution of RiHab scores among participants with TEDs, which appear to be nearly uniformly dispersed across a score range of 3–15, as illustrated in Figure 1.

The original tool was previously assessed in a cohort of 307 participants, who were stratified into a training sub-sample (80%, n = 246) and a test sub-sample (20%, n = 61) [10]. The training sub-sample served as the basis for the development of the scoring algorithm, which achieved an optimal balance between sensitivity and specificity, recording 90% and 72%, respectively, at a cut-off score of four. The cut-off score was further validated in the test sub-sample, demonstrating a sensitivity and specificity of 76% and 80%, respectively. However, it is crucial to note that this original cohort exhibited an extraordinarily high prevalence of TEDs, amounting to 75%. The scoring algorithm was thus developed based on this unrepresentative, high-prevalence sample, which was neither random nor reflective of the broader population of individuals seeking HAs. As such, the generalizability of the tool’s screening accuracy of more representative populations—such as the one investigated in the present study, where the prevalence of TEDs was markedly lower—is questionable, a limitation also reported by the CEDRA developers [10]. As RiHab is intended for use in a RESA environment alongside supplemental audiometric and tympanometric measures as well as visual images of the tympanic membranes, a higher cut-off score might be favored to enhance the tool’s specificity. This would serve to minimize potential costs arising from the evaluation of a greater number of false-positive screenings. While a higher cut-off score would likely reduce RiHab’s sensitivity, the collective sensitivity of the RESA routine could be augmented by the additional diagnostic tests incorporated within the RESA framework.

While the translation and cross-cultural adaptation process followed field-specific guidelines, several potential limitations warrant attention. Direct translation of items might not always grasp the subtleties or cultural nuances of the original content. Additionally, certain concepts in the source culture might lack a direct counterpart in the target culture or may be interpreted differently. Such disparities can lead to variations in item interpretation between the CEDRA and RiHab versions, possibly introducing response biases. To mitigate this, we employed rigorous forward and back translations, coupled with cognitive interviews with 30 intended respondents and reviews by a diverse expert committee. Furthermore, as previously highlighted, incorporating an IRT analysis in future studies could further bolster the validity of the translation. Also, while pilot testing was conducted with 600 respondents, future studies might benefit from a larger sample size. This would account for the uncertain prevalence of TEDs in the target population, thereby enhancing the generalizability of our findings [8,19].

Given the escalating prevalence of age-related HL among individuals aged 60 years and above, there is an increasing urgency to address this public health concern and mitigate the negative consequences of delayed or inadequate HA intervention. Multimodal, digital screening approaches, incorporating elements such as RiHab, have the potential to streamline diagnostic procedures and expedite treatment initiation for this demographic. The implementation of RESA screening for adult first-time HA users presents significant advantages for Denmark and other developed nations where standard diagnostic protocols necessitate an in-person diagnostic process, mitigates extended diagnostic durations, and reduces treatment delays. Furthermore, it holds the potential to optimize socioeconomic resource distribution within the realm of hearing rehabilitation healthcare, all while maintaining patient safety and upholding current examination benchmarks. Nevertheless, the realization of these benefits hinges on the validation of both RiHab and the RESA screening model in a broader, more representative sample for a comprehensive evaluation of the model’s screening accuracy, as well as the efficacy and interrelationship of its constituent components.

## Figures and Tables

**Table 1:** Trade-off of sensitivity and specificity for selected RiHab scores.

RiHab Score	Sensitivity (%)	95% Confidence Interval	Specificity (%)	95% Confidence Interval
2	95	(90–99)	17	(14–21)
3	90	(82–96)	32	(28–36)
4	85	(77–92)	47	(43–51)
5	74	(64–83)	62	(58–66)
6	65	(54–76)	74	(70–77)
7	55	(45–65)	81	(77–84)
8	47	(36–59)	88	(85–90)
9	40	(28–51)	91	(88–93)
10	33	(23–45)	97	(96–99)

**Table 2:** Spearman’s correlation coefficients for individual RiHab items in 113 test–retest respondents.

Item	Spearman’s Correlation Coefficient	95% Confidence Interval	*p*-Value
#1	0.78	0.69–0.84	<0.0001
#2	0.65	0.53–0.74	<0.0001
#3	0.52	0.37–0.74	<0.0001
#4	0.80	0.72–0.86	<0.0001
#5	0.78	0.69–0.84	<0.0001
#6	0.43	0.27–0.57	<0.0001
#7	0.70	0.60–0.79	<0.0001
#8	0.76	0.67–0.83	<0.0001
#9	NA *	NA *	NA *
#10	0.73	0.63–0.81	<0.0001
#11	0.73	0.63–0.81	<0.0001
#12	0.74	0.64–0.81	<0.0001
#13	0.91	0.88–0.94	<0.0001
#13a	0.76	0.61–0.85	<0.0001
#13b-1	0.72	0.61–0.80	<0.0001
#13b-2	0.65	0.53–0.74	<0.0001
#13b-3	0.50	0.35–0.63	<0.0001
#13b-4	0.50	0.35–0.63	<0.0001
#14-1	0.23	0.05–0.40	0.0125
#14-2	0.67	0.55–0.76	<0.0001
#15-1	NA *	NA *	NA *
#15-2	NA *	NA *	NA *
#15-3	0.66	0.54–0.75	<0.0001
#15-4	0.41	0.24–0.55	<0.0001
#15-5	0.59	0.46–0.70	<0.0001
#15-6	0.74	0.64–0.81	<0.0001
#15-7	0.70	0.60–0.79	<0.0001
#15-8	0.66	0.55–0.76	<0.0001
#15-9	0.77	0.68–0.83	<0.0001

* As all 113 respondents scored the exact same on the test and the retest for items 9, 15-1, and 15-2, the correlation coefficients were not calculated for these items due to lack of variability. NA indicates not available.

## Data Availability

In pursuance of Danish legislation, data will be made available only after approval from the Danish Data Protection Agency and after signing an access agreement.

## Supplementary Materials

The following supporting information can be downloaded at: https://www.mdpi.com/article/10.3390/audiolres13060075/s1. Risikovurdering af Høreapparatbrugere (RiHab).
