# Supplementary material for: Cross-Cultural Translation and Adaptation of the Consumer Ear Disease Risk Assessment (CEDRA) Questionnaire in Danish
Source: Audiol Res. 2023 Nov 2;13(6):859–70. doi: 10.3390/audiolres13060075 (PMC10660472; doi:10.3390/audiolres13060075)
Supplement: Supplementary file 1 [file audiolres-13-00075-s001.zip › Supplementary Material (RiHab).pdf]

Dette spørgeskema er lavet med henblik på at hjælpe dig med at vurdere, om du bør ses af en læge forud for behandling med høreapparat. Hvis du har helbredsmæssige spørgsmål eller bekymringer angående din hørelse, bør du opsøge en læge uanset udfaldet af dette spørgeskema.

## Spørgsmål angående dine ører og din hørelse

***Sæt ring om "Ja" eller "Nej"***

| | | |
|---|---|---|
| 1. Når du taler i telefon, forstår du så bedre, hvad der bliver sagt med det ene øre end med det andet? | Ja | Nej |
| 2. Er hørenedsættelsen på det ene eller begge ører opstået pludseligt? | Ja | Nej |
| 3. Har du tidligere oplevet en pludseligt opstået, blivende hørenedsættelse? | Ja | Nej |
| 4. Oplever du hørenedsættelse på kun ét øre? | Ja | Nej |
| 5. Hører du bedre med det ene øre end med det andet? | Ja | Nej |
| 6. Ændrer din hørelse sig fra dag til dag? | Ja | Nej |
| 7. Har du som voksen haft infektion i det samme øre mere end én gang i løbet af ét år? | Ja | Nej |
| 8. Har du nogensinde oplevet, at det flyder med betændelse, blod eller anden væske fra dit øre? | Ja | Nej |
| 9. Har du nogensinde fået stillet diagnosen Ménières sygdom af en læge? Hvis du aldrig har hørt om Ménières sygdom, skal du sætte ring om "Nej". | Ja | Nej |

10. Hvordan vil du generelt vurdere dit helbred?
- ☐ Meget godt
- ☐ Godt
- ☐ Dårligt
- ☐ Meget dårligt

11. Hvor ofte er du svimmel?
- ☐ Aldrig
- ☐ Ind i mellem
- ☐ Ofte
- ☐ Altid

12. Hvordan vurderer du din balance?
- ☐ Meget god
- ☐ God
- ☐ Dårlig
- ☐ Meget dårlig

13. Har du tinnitus i form af en ringende, susende eller klikkende lyd i ørerne? | Ja | Nej

***Hvis du har sat ring om "Nej", gå videre til spørgsmål 14.***

13a. Hvis du har sat ring om "Ja" i spørgsmål 13: Har du tinnitus i (sæt kun ét kryds):

- ☐ Højre øre
- ☐ Venstre øre
- ☐ Begge ører
- ☐ Ved ikke

13b. Hvis du har sat ring om "Ja" i spørgsmål 13, har du i så fald ét eller flere af de følgende symptomer sammen med din tinnitus?

| | | |
|---|---|---|
| Svimmelhed | Ja | Nej |
| Trykfornemmelse i øret, eksempelvis som hvis noget trykker eller presser i øret eller øregangen | Ja | Nej |
| Fyldefornemmelse i øret, eksempelvis som hvis noget optager plads i øret eller øregangen | Ja | Nej |
| Propfornemmelse i øret, eksempelvis som hvis noget blokerer for lyden i øret eller øregangen | Ja | Nej |

14. Har du nogensinde haft ét eller flere af de følgende symptomer i mere end 10 minutter?

| | | |
|---|---|---|
| Pludselig hørenedsættelse på ét eller begge ører | Ja | Nej |
| Pludselig synsændring på ét eller begge øjne | Ja | Nej |

15. Har du inden for de sidste 3 måneder haft ét eller flere af følgende symptomer?

| | | |
|---|---|---|
| Vedvarende flåd fra ét eller begge ører | Ja | Nej |
| Betændelse eller blod fra ørerne | Ja | Nej |
| Vedvarende smerter i eller omkring ørerne | Ja | Nej |
| Ændring af hørelsen på et eller begge ører | Ja | Nej |
| Forkølelse eller bihuleproblemer, der forværrede din hørelse | Ja | Nej |
| Svimmelhed | Ja | Nej |
| Fald pga. dårlig balance | Ja | Nej |
| Vedvarende eller tilbagevendende hovedpine | Ja | Nej |
| Tilbagevendende feber, nattesved eller kulderystelser? | Ja | Nej |

## Resultatark

*Du skal, inden du beregner dit resultat, svare på alle spørgsmål på side 1 og 2. Når du har sikret dig, at du har besvaret alle 15 spørgsmål på side 1 og 2, kan du fortsætte med at beregne dit resultat.*

Følg instruktionen og angiv point for spørgsmålene 1-9 og 10-15:

| Spørgsmål | | Point |
|---|---|---|
| 1 | **Èt** point, hvis du har sat ring om "Ja". **Nul** point ved "Nej" | |
| 2 | **Èt** point, hvis du har sat ring om "Ja". **Nul** point ved "Nej" | |
| 3 | **Ét** point, hvis du har sat ring om "Ja". **Nul** point ved "Nej" | |
| 4 | **Ét** point, hvis du har sat ring om "Ja". **Nul** point ved "Nej" | |
| 5 | **Ét** point, hvis du har sat ring om "Ja". **Nul** point ved "Nej" | |
| 6 | **Ét** point, hvis du har sat ring om "Ja". **Nul** point ved "Nej" | |
| 7 | **Ét** point, hvis du har sat ring om "Ja". **Nul** point ved "Nej" | |
| 8 | **Ét** point, hvis du har sat ring om "Ja". **Nul** point ved "Nej" | |
| 9 | **Ét** point, hvis du har sat ring om "Ja". **Nul** point ved "Nej" | |
| | *Læg pointene sammen for spørgsmålene 1-9* ***[A]:*** | |

| Spørgsmål | | Point |
|---|---|---|
| 10 | **Ét** point hvis du har sat kryds ved "Dårligt" eller "Meget dårligt" | |
| 11 | **Ét** point hvis du har sat kryds ved "Ofte" eller "Altid" | |
| 12 | **Ét** point hvis du har sat kryds ved "Dårlig" eller "Meget dårlig" | |
| 13 | **Ingen point** for dette spørgsmål | 0 |
| 13a | **Ét** point hvis du har sat kryds ved **enten** "Højre øre" **ELLER** "Venstre øre"<br>**Nul** point hvis du har sat kryds ved **begge** | |
| 13b | Antallet af gange du har svaret "Ja" | |
| 14 | Antallet af gange du har svaret "Ja" | |
| 15 | Antallet af gange du har svaret "Ja" | |
| | *Læg pointene sammen for spørgsmålene 10-15* ***[B]:*** | |

Læg pointene ovenfra sammen: **[A]** + **[B]** = RiHab-resultat: __________

Hvis dit samlede resultat er **4** eller derover, bør du tale med en læge om dine symptomer.
